# The urethro-prostatic angle as a practical sonographic index for estimating prostatic size in dogs

**DOI:** 10.3389/fvets.2025.1753119

**Published:** 2026-01-30

**Authors:** Debora Groppetti, Alessandro Pecile, Elisa Giussani, Valerio Bronzo, Federica Raneri, Stefano Faverzani

**Affiliations:** 1 Department of Veterinary Medicine and Animal Sciences, Università degli Studi di Milano, Milan, Italy; 2 Clinica Veterinaria Mengotto, Tradate, Italy

**Keywords:** angle, canine (dog), measurement, prostatomegaly, ultrasound

## Abstract

**Introduction:**

Prostatomegaly is a common condition in adult, intact dogs. Abdominal ultrasonography is currently considered the method of choice for assessing the prostate size using volume-based formulas. However, objectively defining an enlarged prostate remains challenging, even for experienced operators. This is mainly due to the wide variability in dog sizes and the partially intrapelvic position of the gland, which can limit its complete visualization. This study aimed to explore the reliability of a new, practical, and less operator-dependent parameter, the urethro-prostatic angle (UPa), for estimating canine prostate volume via B-mode ultrasonography.

**Materials and methods:**

Sixty-three dogs were enrolled, and prostatic volume was assessed using the equation proposed by Ruel et al., which is based on measurements of the gland's length, height, and width obtained from sagittal and transverse scans. Furthermore, to distinguish between normal and enlarged prostates, a second Ruel formula, which includes age and body weight, was also applied.

**Results:**

The urethro-prostatic angle was successfully measured in all dogs and correlated significantly with prostate volume (*p* < 0.001). A UPa cutoff value of 96.52° effectively discriminated between normal (UPa ≤ 96.52°) and enlarged (UPa > 96.52°) prostates, achieving a sensitivity of 100% and a specificity of 71.7%. Furthermore, inter-operator variability was not significant (*p* = 0.2).

**Conclusion:**

In contrast to traditional volume estimation methods, the measurement of the UPa is independent of canine body size, prostatic gland position, and operator skill. These findings suggest that the UPa is a promising, repeatable, and practical parameter for the reliable estimation of prostatomegaly in dogs.

## 1 Introduction

Many pathological conditions can lead to prostatic enlargement in dogs, including benign prostatic hyperplasia (BPH), prostatitis, neoplasia and squamous metaplasia (1). BPH is considered an almost ubiquitous condition in aging intact male dogs, with a prevalence ranging from approximately 46% to as high as 90% by 8 years of age (2, 3). Prostatitis is also relatively common (38.5%), whereas prostatic neoplasia (0.5%) and squamous metaplasia (0.2%) are rare (4, 5). Prostatitis, particularly acute form, may progress to abscess formation (7.7%), which together with large intraprostatic cysts and neoplasia can lead to deformation of the gland's margins (1, 5). The conventional approach for estimating canine prostate size relies on the measurement of three diameters (length, height, and width) obtained from longitudinal and transverse transabdominal ultrasound views (6–10). Although widely adopted, this method does not adequately account for anatomical variability and may be susceptible to errors and operator-dependent bias. This is partly due to the considerable heterogeneity in body size among dogs, as prostatic dimensions have been shown to correlate with body weight (11). Moreover, accurate measurement can be hindered by the anatomical position of the prostate, particularly when it is partially or fully located within the pelvic cavity, to which bladder repletion also contributes (12). Furthermore, both the operator's experience and the ultrasound equipment can significantly influence measurement accuracy, further compromising the reliability of volumetric estimations (13). Therefore, defining canine prostate enlargement remains a clinical challenge.

In light of these limitations, we considered the urethro-prostatic angle (UPa) as an alternative parameter, given that it is not influenced by body size and remains detectible even when the caudal pole of the prostate is intrapelvic. For this purpose, the UPa was measured alongside the length, height, and width of the prostate, and its value was compared to the prostatic volume calculated according to the literature (6). Furthermore, to assess its clinical utility in distinguishing between normal and enlarged prostates, the UPa was correlated with the Ruel's formula, which estimates expected gland volume based on age and body weight (6). Due to the known tendency of the hyperplastic prostate to develop multiple small cysts (14–16), the angle was also analyzed with respect to the gland echotexture. The repeatability and ease of application of the UPa in routine practice were assessed by comparing measurements obtained from an expert and a beginner ultrasonographer.

## 2 Materials and methods

### 2.1 Animals

For this study, we included all prostatic images obtained from intact male dogs referred to the Veterinary Teaching Hospital at the University of Milan, regardless of the animals' general health status or specific prostatic condition. Inclusion required the availability of both longitudinal and transverse scan planes, as well as ultrasound measurements of prostatic length (L), height (H), and width (W). Cases were excluded if any pathological process was present that could distort the visualization of the urethra. A total of 63 adult intact male dogs, both purebred and mixed-breed, aged from 1 to 15 years (6.9 ± 3.7) and weighing from 5.4 to 65 kg (27.9 ± 13.9), were enrolled.

### 2.2 Ultrasonographic protocol

Ultrasound examinations were performed with a distended bladder, and the dogs were positioned either in lateral recumbency or standing. When necessary, the para-penile area was clipped to optimize prostate visualization, and alcohol and ultrasound gel were applied to ensure effective probe-to-skin contact. For each dog, both sagittal and transverse B-mode ultrasound images of the prostate were obtained using a MyLab X90-Vet Esaote^®^ equipped with a multifrequency micro-convex probe (3–11 MHz) and a multifrequency linear probe (4–20 MHz). Ultrasound frequency settings were tailored based on the individual physical characteristics of the patient.

### 2.3 Measurement technique

**Figure 1:**
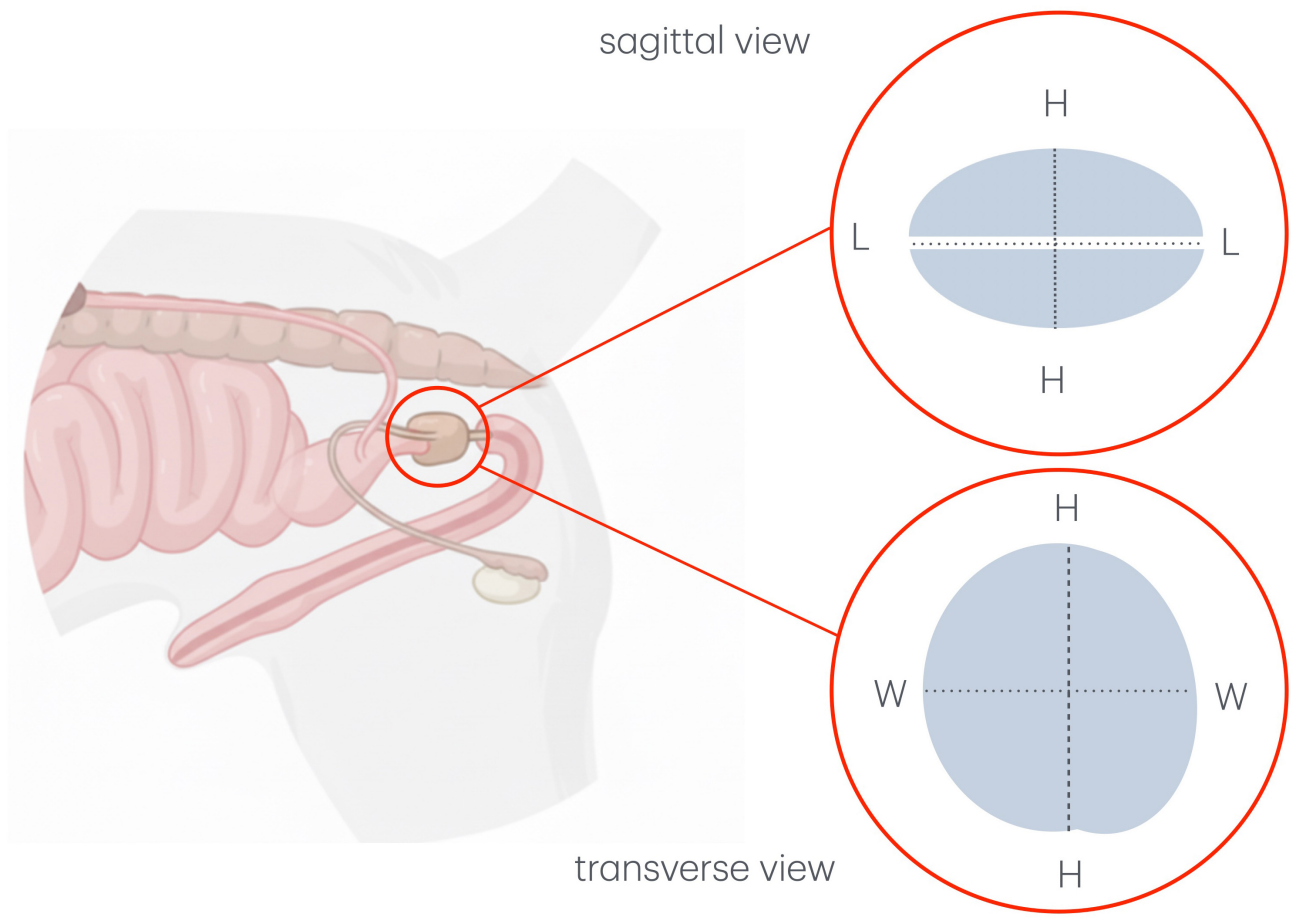
Schematic representation showing sagittal and transverse measurements of the canine prostate. “L” represents prostatic length; “H” represents prostatic height; “W” represents prostatic width. This figure has been partially generated with the support of BioRender.x.

**Figure 2:**
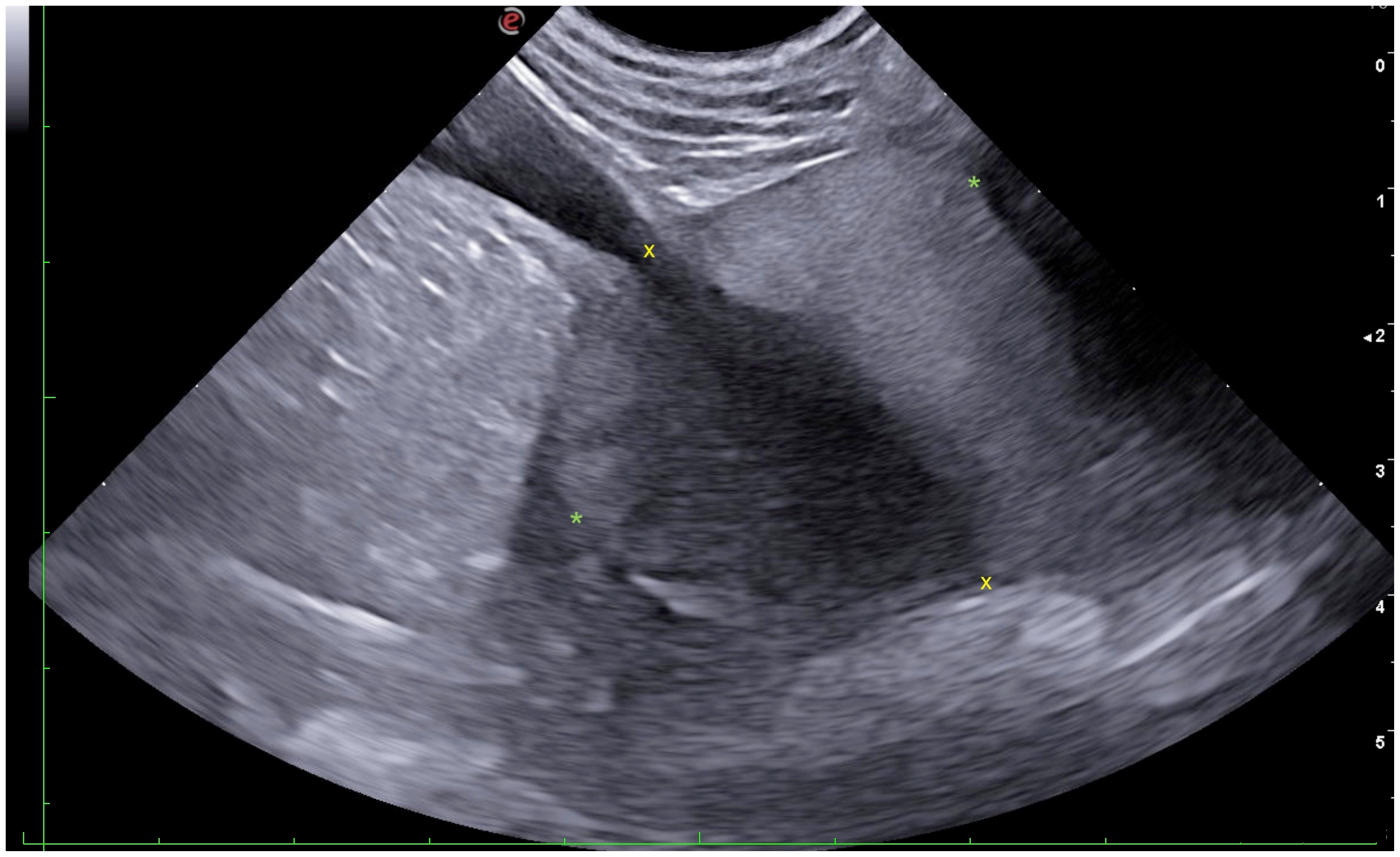
Sagittal ultrasonographic section of the canine prostate demonstrating measurement methodology. Yellow “x” marks outline the measurement of prostatic length (L); green “*” marks outline the measurement of prostatic height (H).

**Figure 3:**
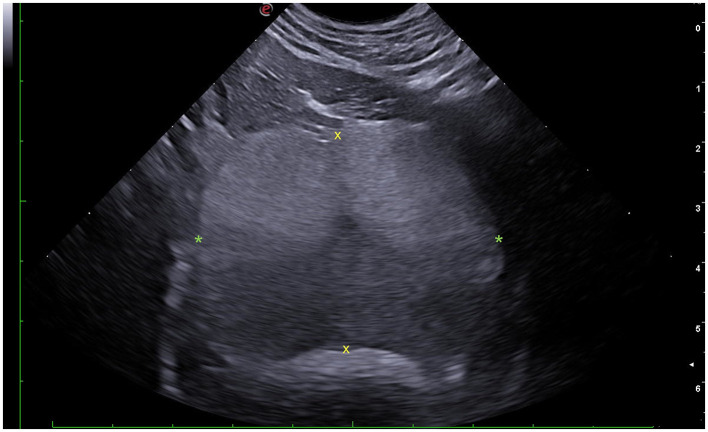
Transverse ultrasonographic section of the canine prostate demonstrating measurement methodology. Yellow “x” marks outline the measurement of prostatic length (L); green “*” marks outline the measurement of prostatic width (W).

Prostatic length (L) was measured in the sagittal plane as the distance between the cranial and caudal pole along the urethral axis (Figures 1, 2). Height (H) was assessed in both sagittal and transverse planes: in the sagittal plane as the maximum diameter perpendicular to the urethral axis (Figures 1, 2), and in the transverse plane as the maximum distance between the dorsal and ventral surfaces of the gland, on a line separating the two lobes and passing through the urethra (Figures 1, 3). Width (W) was measured in the transverse plane as the greatest diameter perpendicular to the line of the height and passing through the urethra (Figures 1, 3). Each parameter was calculated as the average of three consecutive measurements, with H expressed as the mean of the sagittal and transverse values (6).

**Figure 4:**
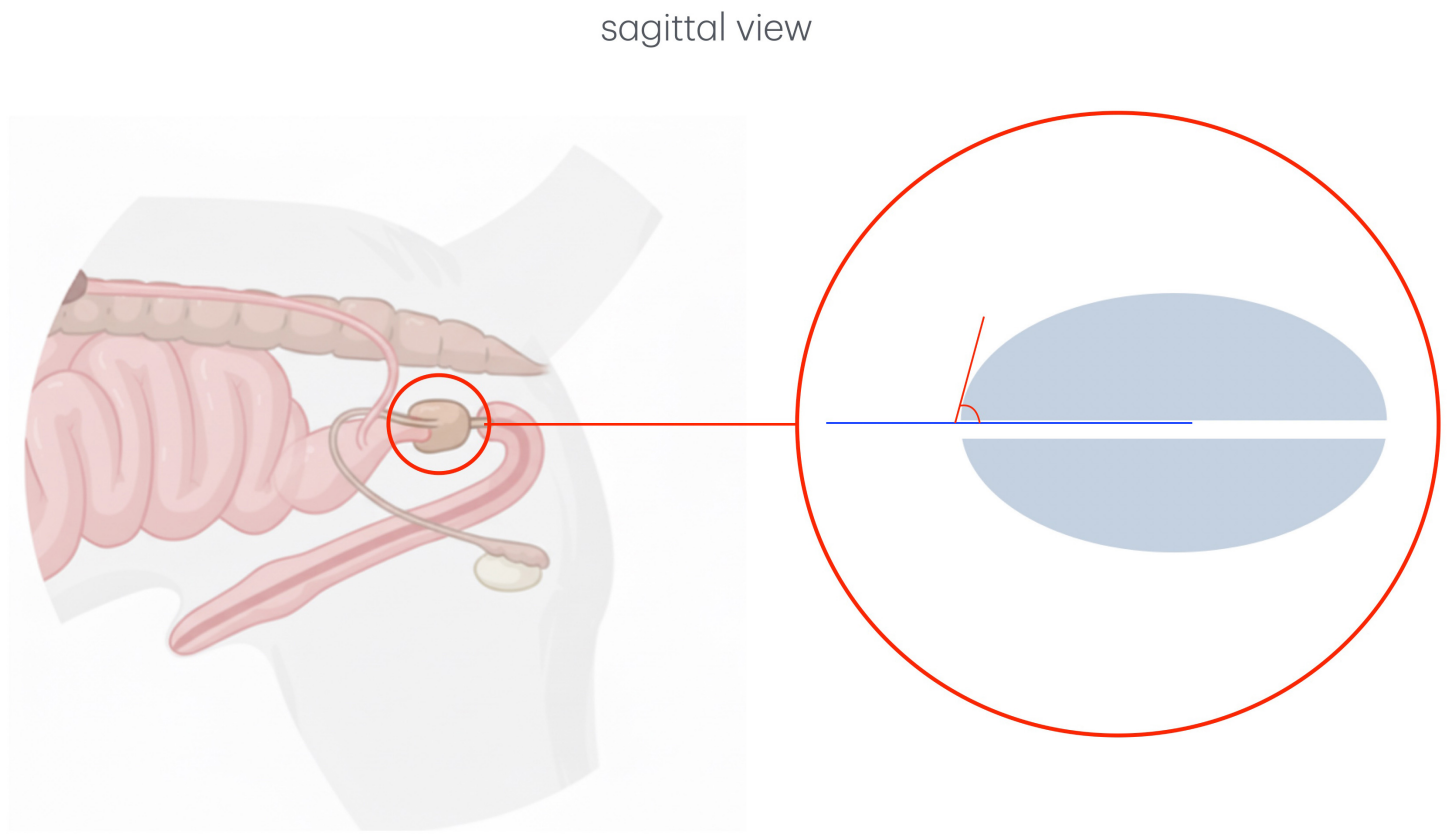
Schematic representation of the urethro-prostatic angle measurement. This figure has been partially generated with the support of BioRender.x.

In the sagittal plane, the UPa was also measured. This angle is defined by the intersection between the urethral axis and the line tangent to the cranial pole of the prostate, passing through the junction between the prostate and the urethra (Figure 4).

Measurements for prostatic length, height, width and UPa amplitude were performed with the MedDream version 8.3.0-dev software (Softneta^^®^^, UAB, Saronno, Italy).

The value of the UPa was correlated to the estimated prostatic volume calculated using the ellipsoid volume formula (6).


$$\begin{array}{l} volume\;=\;length\;\times \;width\;\times \;height\;\times \;0.523 \end{array}$$ (1)

An enlarged prostate was defined as a prostate volume greater than the value predicted by the Ruel Equation 2, which accounts for age and body weight (6).


$$\begin{array}{l} maximum\;volume\;=\;\left(0.867\;\times \;body\;weight\right)\;+\;\left(1.885\;\times \;age\right)\;\\ \;+\;15.88\;\left(2\right) \end{array}$$

The predictive capacity of the UPa for discriminating between normal and enlarged prostates was investigated, based on the volume calculated using the Ruel Equation 2.

In addition, the UPa was assessed against prostatic echotexture. Specifically, the glands were classified according to their parenchymal homogeneity (defined as a uniform, smooth, and finely granular appearance) or heterogeneity (defined by coarse granularity with hypoechoic or hyperechoic focal areas).

The prostate volume and UPa measurements were performed by two independent and blinded operators with different levels of experience, and the results were compared to assess inter-operator variability. All measurements were conducted on the same acquired ultrasound images. This approach was chosen to eliminate confounding effects arising from the inherent differences in probe positioning between distinct acquisition, which could have otherwise skewed the outcomes.

### 2.4 Statistical analysis

Statistical analyses were performed using IBM SPSS Statistics for Windows, version 29.0 (IBM Corp., Armonk, NY, USA). Given the non-normal distribution of the data, non-parametric correlation Rho Spearman was used to compare the UPa value with prostatic volume, calculated with Ruel Equation 1. Rho Spearman's rank correlation was also used to examine the relationships between the UPa and age and body weight. Receiver Operating Characteristic (ROC) curve analysis was performed to evaluate the ability of UPa to discriminate between small and large prostate volumes, as categorized using the Ruel Equation 2. The area under the curve, along with its 95% confidence interval (CI), standard error, and significance level, were computed. The Youden index was used to identify the optimal cut-off value for the angle, and sensitivity and specificity were assessed at various threshold levels. Non-parametric Mann–Whitney *U* test was applied to compare ultrasound operators. Pairwise comparisons were adjusted using the Bonferroni correction for multiple comparisons. All tests were two-tailed, with a significance threshold set at *p* < 0.05.

## 3 Results

The prostatic volume calculated using the Ruel Equation 1 had a mean of 36.17 ± 35.08 cm^3^ (2.45–165.32). The maximum prostatic volume calculating by the Ruel Equation 2 was 52.7 ± 13.4 cm^3^ (13.4–89.2).

According to the Ruel Equation 2, 11 dogs out of 63 (17.5%) had an enlarged prostate. Homogeneous prostatic parenchyma was observed in 44 dogs (69.8%), and heterogeneous in 19 (30.1%). None of the dogs was diagnosed with prostatic neoplasia or abscesses.

**Figure 5:**
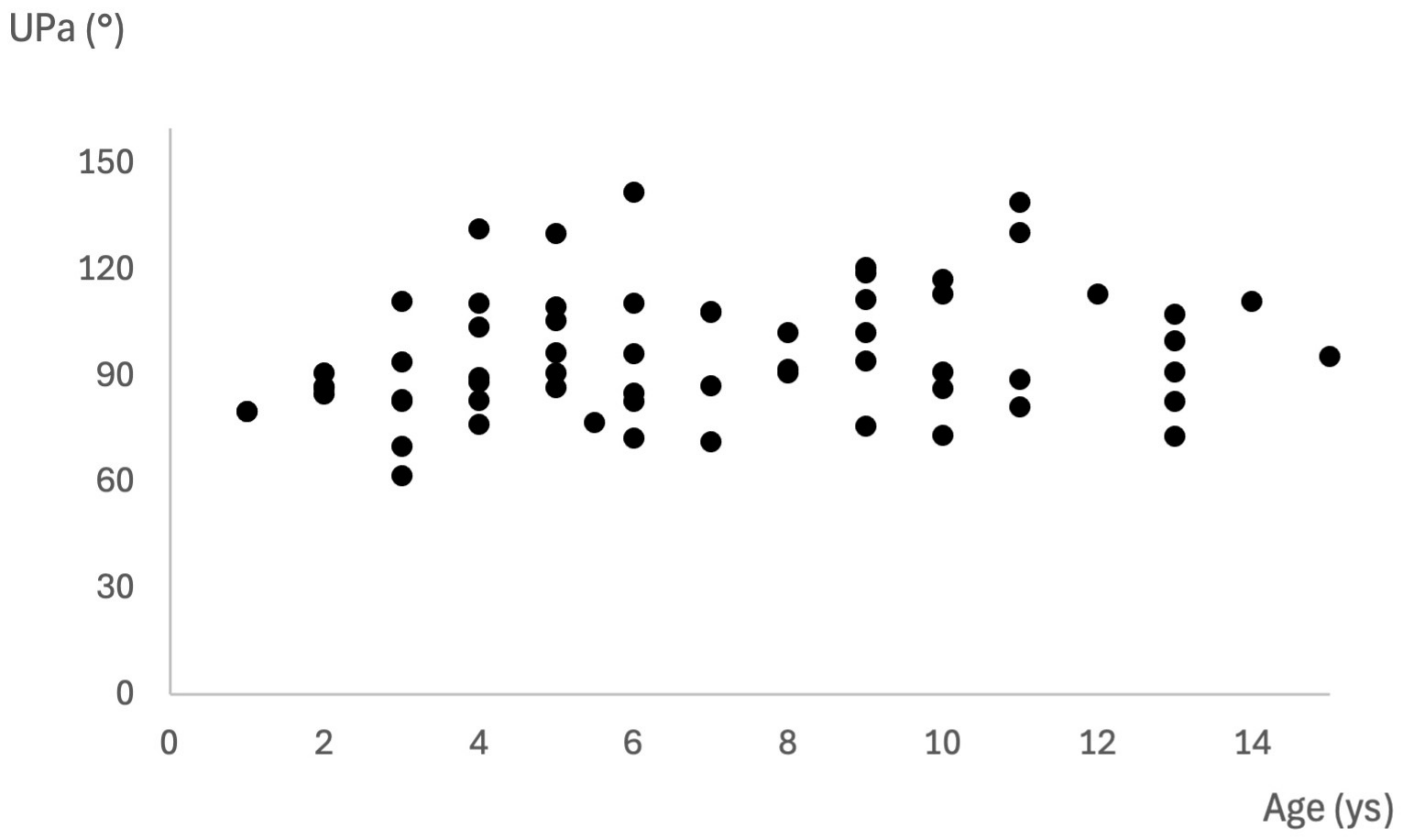
Distribution of UPa values across different ages. Each point corresponds to the UPa value measured in a single dog.

In all 63 dogs, it was possible to measure the UPa, which was 96.1 ± 17.7 degrees (61.7–141.9). Age (*p* = 0.03) but not body weight (*p* = 0.37) was correlated with the UPa (Figure 5).

The prostatic volume (Ruel Equation 1) was positively correlated to the UPa (*p* < 0.001).

**Figure 6:**
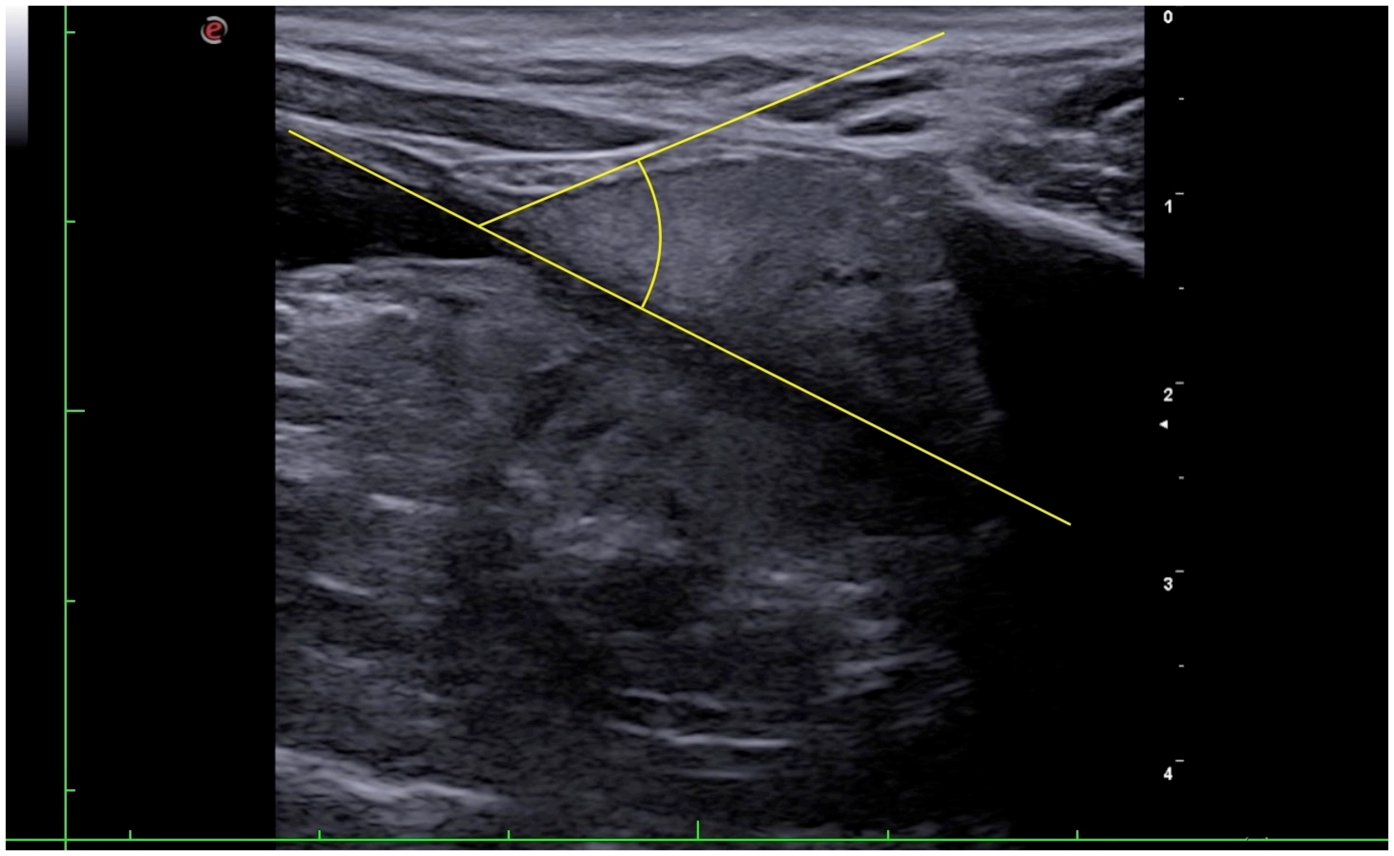
Sagittal ultrasonographic view of a canine prostate with normal volume. The yellow lines indicate the urethro-prostatic angle.

**Figure 7:**
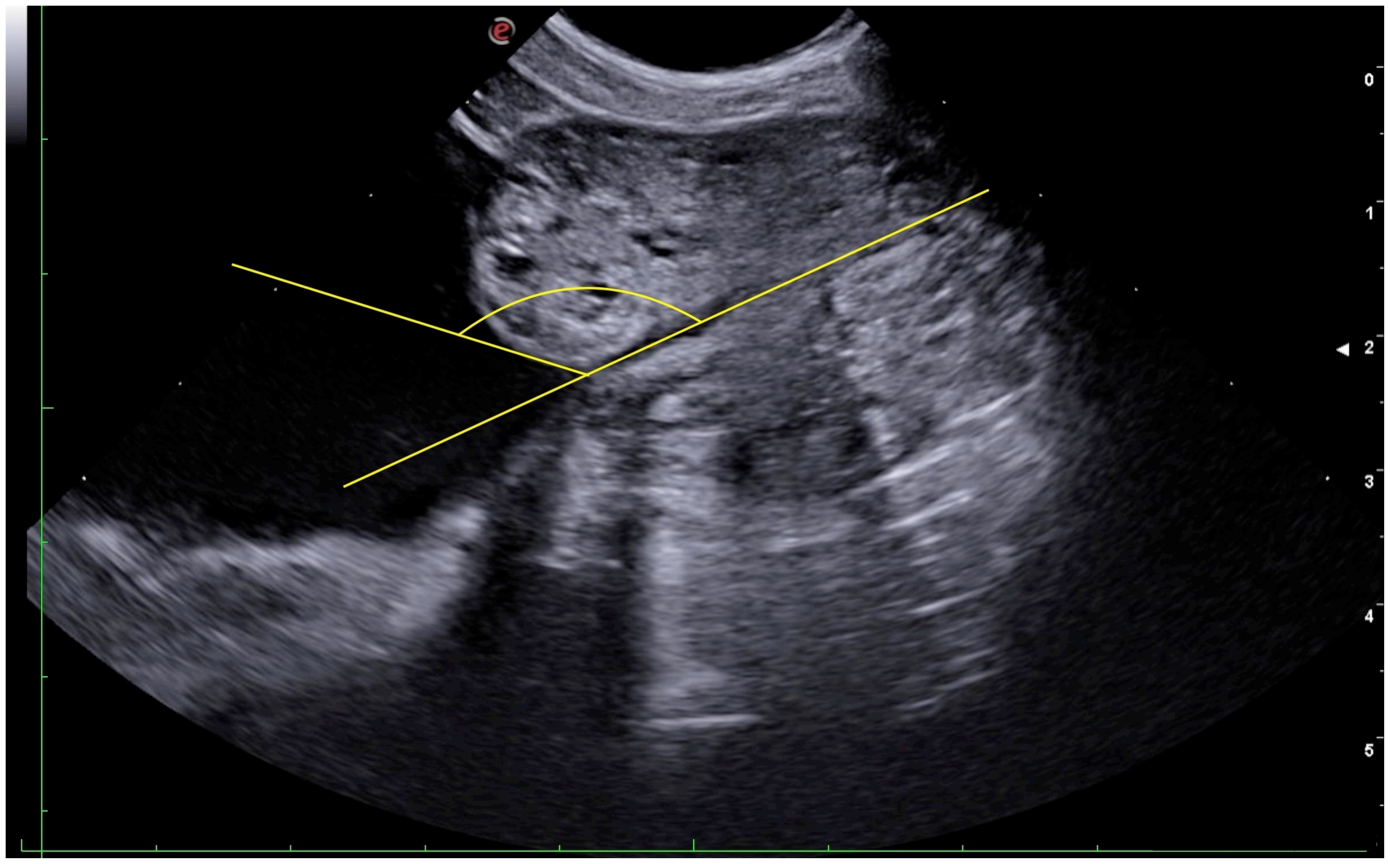
Sagittal ultrasonographic view of a canine prostate with enlarged volume. The yellow lines indicate the urethro-prostatic angle.

**Figure 8:**
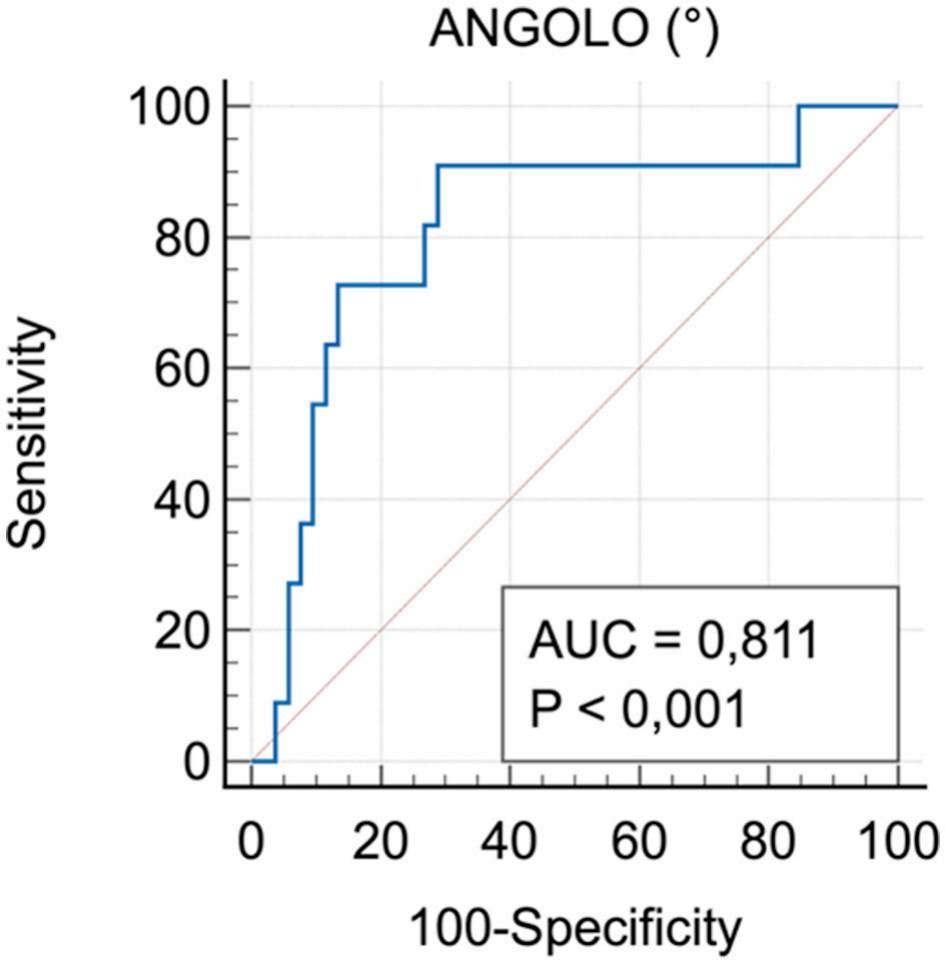
Diagnostic performance of the UPa measurement to discriminate between normal and enlarged prostates. The red line is the reference, while the blue represents the ROC curve of the UPa measurement.

The UPa threshold of 96.52° allowed discriminating between normal (UPa ≤ 96.52°) and enlarged (UPa > 96.5°) prostate based on the Ruel Equation 2, with sensitivity of 100% and specificity of 71.7 % (*p* < 0.001; Figures 6–8).

A significant difference in UPa amplitude was observed according to the prostatic echotexture. Dogs with parenchymal heterogeneity (*p* < 0.001) showed higher UPa values compared to those with a normal homogeneous prostatic ultrasound appearance.

Finally, no significant differences were found between the measurements of the UPa performed by the two operators (*p* = 0.2).

## 4 Discussion

The urethro-prostatic angle (UPa) has previously been evaluated in human medicine as a parameter associated with lower urinary tract symptoms and benign prostatic hyperplasia (17–20). To the best of our knowledge, this parameter has not yet been described or applied in dogs, and no veterinary studies have investigated its diagnostic value in canine prostatic disorders. This study represents the first adaptation and evaluation of the urethro-prostatic angle (UPa) in the canine species. Despite the necessary distinctions arising from anatomical differences, posture, and ultrasound access routes (transrectal in humans vs. transabdominal in dogs), this technique also shows promising results in the canine species. In fact, UPa measurement was feasible and easily performed in all dogs enrolled in this study, even when only the cranial pole of the prostate was visible on ultrasound. An important and original aspect of this strategy lies in its geometric nature: the UPa is a physical parameter, inherently independent of individual factors. Unlike traditional linear or volumetric measurements, which require adjustment or correction factors to account for individual variability, the UPa is scalable and adaptable regardless of the dog's size. Indeed, prostate size is known to positively correlate with dog size (21–23). An aspect of particular importance in dogs, given the marked morphotype variability observed among breeds. Notably, the angle amplitude showed no correlation with body weight in the dogs assessed, confirming that it is not influenced by breed or body condition. This contrasts with other methods, such as normalizing prostatic dimensions to the aortic diameter or body weight or age, which have been proposed to reduce inter-individual variation (6, 7, 11).

For this reason, along with the objective difficulty in precisely delimiting the gland's margins in this species, defining prostate enlargement remains a clinical challenge, even though several equations have been proposed to calculate prostatic volume using transabdominal ultrasound measurements (6–10). Our results showed a significant correlation between UPa and estimated prostatic volume (Ruel Equation 1), supporting the value of this angle as an indirect marker of prostatic enlargement. Moreover, we identified a cutoff value that effectively discriminates between normal ( ≤ UPa 96.52°) and increased (> UPa 96.52°) prostate size (Ruel Equation 2) (6). The establishment of a clinically relevant cut-off offers the potential for a simple, non-invasive utility across several clinical domains, including patient screening, post-therapy follow-up, and the facilitation of differential diagnosis.

The most common cause of prostate enlargement in dogs is benign prostatic hyperplasia (BPH).

Although often asymptomatic, BPH can compromise fertility, with relevant implication for breeding animals, and can also result in clinical signs such as hematuria, dysuria, and tenesmus (5). The diagnosis of BPH mainly relies on the assessment of prostate shape, size, and echogenicity, as well as the measurement of canine prostatic-specific esterase (CPSE) levels (23, 24). To date ultrasonography is the recommended non-invasive method for evaluating the canine prostate gland and estimating its dimension (6, 7, 10, 25, 26). BPH typically develops in dogs over 5 years of age. Conversely, a reduction in gland size, likely due to senile involution, may occur in dogs older than 7 or 11 years, depending on breed-specific life expectancy (9, 14, 27). In agreement with this evidence, the age of dogs in our caseload influenced the UPa amplitude. Besides an increase in prostate volume, BPH is characterized by heterogeneous ultrasound appearance with multiple intraparenchymal cysts (miliary to large), which may be either hypoechoic or anechoic on the image (16, 28, 29). UPa was also correlated to prostate echotexture, further confirming its value as a promising diagnostic indicator of BPH. Nevertheless, given that multiple factors may contribute to prostatic enlargement, the association between BPH and the angle amplitude requires further validation.

Finally, the low inter-operator variation observed in this study underlines the repeatability and ease of use of this technique, which does not require advanced training. Calculating the angle from pre-acquired scans rather than from images obtained directly by each operator introduces a limitation related to potential variability in probe positioning, as could occur under realistic clinical conditions. However, acquiring the UPa value requires only obtaining a longitudinal scan of the prostate, which represents the minimal level of skill expected even from an inexperienced operator.

We acknowledge some constraints to this study, specifically the heterogeneity of the sample and the limited generalizability due to the lack of data from inter-operator image acquisition.

As a planned extension of this study, we intend to refine the UPa assessment using real-time images acquired by multiple operators. Moreover, we will investigate its potential correlation with different prostatic conditions, biochemical markers (e.g., CPSE, alkaline phosphatase), and sperm quality to further elucidate its diagnostic role.

## 5 Conclusions

These findings suggest that UPa may serve as a useful tool for early detection of prostatomegaly in dogs, offering promising complementary support to CPSE in BPH diagnosis and monitoring. The simplicity of UPa measurement makes it particularly suitable for general clinical practice and screening purposes. The use of an angle, as opposed to absolute dimensions, could represent a novel and more standardized perspective in the ultrasonographic assessment of the canine prostate.

## Data Availability

The raw data supporting the conclusions of this article will be made available by the authors, without undue reservation.
